# Knocking and Listening: Learning Mechanical Impulse Response for Understanding Surface Characteristics

**DOI:** 10.3390/s20020369

**Published:** 2020-01-09

**Authors:** Semin Ryu, Seung-Chan Kim

**Affiliations:** Intelligent Robotics Laboratory, Hallym University, Chuncheon 24252, Korea; sr@hallym.ac.kr

**Keywords:** context understanding, time series classification, mechanical impulse, sequence learning

## Abstract

Inspired by spiders that can generate and sense vibrations to obtain information regarding a substrate, we propose an intelligent system that can recognize the type of surface being touched by knocking the surface and listening to the vibrations. Hence, we developed a system that is equipped with an electromagnetic hammer for hitting the ground and an accelerometer for measuring the mechanical responses induced by the impact. We investigate the feasibility of sensing 10 different daily surfaces through various machine-learning techniques including recent deep-learning approaches. Although some test surfaces are similar, experimental results show that our system can recognize 10 different surfaces remarkably well (test accuracy of 98.66%). In addition, our results without directly hitting the surface (internal impact) exhibited considerably high test accuracy (97.51%). Finally, we conclude this paper with the limitations and future directions of the study.

## 1. Introduction

In nature, several insects, such as treehoppers, sawflies, and butterflies, are capable of communicating using vibrations to obtain information regarding their surroundings [1,2]. For example, sawfly larvae can assess the presence of nearby individuals by tapping on a part of the colony, and treehopper nymphs can locate a new feeding site by listening to the vibrations generated by other individuals [2]. Similarly, spiders can recognize preys and even deformities in their web through self-generated vibrations [3,4]. Meanwhile, humans often estimate material properties based on the tactual feeling of tapping on a wall, floor, etc. For example, we tend to knock on a wall to identify its characteristics. In another case, we knock on a watermelon to assess whether the fruit is ripe. Undoubtedly, these tactual approaches help us to obtain additional information that is statistically difficult to understand. The goal of this study is to explore the feasibility of knocking and sensing in daily smart commodities, such as AI (artificial intelligence) speakers. For example, if such devices can inform itself where it is located (e.g., bedroom, kitchen, living room, etc.), it can provide users with a service that is more contextual. If it is located in a kitchen, it can actively provide services related to food cooking, for instance. Although the same purpose can be achieved using camera, privacy issues exist. In fact, a number of studies have been conducted to understand inertial and vibrational signals incoming to a device, as such signals can describe a variety of contextual information. For example, mechano-sensing units embedded in electronic devices, which respond to incoming mechanical stimuli, can differentiate among types of touches [5], gestures on a surface [6], amount of force [7], etc. Touch & Active [8] utilized mechanical vibrations propagated through rigid surfaces to understand the rich context of user touches on daily objects or the configuration of the objects. Although the approaches recognize object characteristics successfully, their application is restricted to objects that generate vibrations or are instrumented with sensing systems.

As humans can identify the characteristics of a surface by knocking on it, we herein propose extending mechano-sensing applications by incorporating knocking strategies such that new smart devices can be developed. Similar to knocking, our system attempts to understand surfaces by creating a mechanical impact on a surface using a push–pull solenoid structure, which comprises an armature (a metal slug) with a coil of wire wrapped around it. The captured signals, i.e., mechanical impulse response, are then analyzed using various machine-learning techniques including recent deep-learning approaches. Ideally, the impulse response contains the system’s response to every frequency component; therefore, it helps the system to better understand the intrinsic characteristics of an object. We believe that the proposed method can extend the dimension of information regarding the surroundings.

In summary, we propose a novel and compact system that, when placed on a surface, can identify it by sensing inertial signals caused by knocking the surface. Furthermore, we demonstrate the feasibility of the approach by conducting a series of experiments. Results suggest that the proposed method can enable context-based interaction based on the location.

## 2. Related Work

### 2.1. Surface Identification

Various methods have been investigated to identify the properties of surfaces, e.g., type, thickness, flaw or defection, and quality. To identify the quality or defection of surfaces, visual inspection based on surface textures and patterns has been widely performed [9,10,11,12,13]. Based on optical reflection, several researchers have detected the surface type by measuring reflected light, which depends on the surface type, from the test surface [14,15,16]. Despite the capability to successfully identify various surfaces, these methods have several limitations. In particular, the performance of visual approaches highly depends on the lighting conditions and angle. Furthermore, in the case of optical reflection-based approaches, the color of the test surface affects the measurements significantly. Tamas et al. [17,18] successfully classified surface properties based on the measurement of thermal response to laser excitation. However, this method may require relatively expensive equipment for application in smart devices. Although a similar purpose can be achieved by measuring the magnetic property of the surface [19], this method is only valid for ferrous materials. Tarapata et al. [20] identified the types of surfaces using ultrasonic waves. They used multiple piezoelectric transducers—one to generate and others to receive ultrasonic signals. Depending on the type of surface, different measurements for the absorption, diffusion, and reflection characteristics of ultrasonic waves could be achieved. However, this method could mostly identify only two types of surfaces—solid and soft. In this study, using a low-cost system, we aim to perform not only the classification between solid and soft surfaces but also the fine-grained classification within solid/soft surfaces.

### 2.2. Vibration-Based Approaches

Many studies have been performed to understand inertial and vibrational signals incoming to a device, as such signals contain a variety of information. For example, mechano-sensing units embedded in electronic devices, which respond to incoming mechanical stimuli, can differentiate among types of touches [5], gestures on a surface [6], amount of force [7], etc. Touch & Active [8] utilized mechanical vibrations propagated through rigid surfaces to understand the rich context of user touches on daily objects or the configuration of the objects. A recent study demonstrated state-of-the-art performances in probabilistic earthquake detection and location based on a convolutional neural network (CNN) using ground waveform data [21]. Early pioneering studies regarding the utilization of generating and sensing strategies focused on electrical signals for recognizing daily objects and interaction types [22]. Although the approach recognizes object characteristics successfully, it is not applicable when the object is not electrically conductive. Furthermore, as the above methods require every object of interest to be equipped with sensors and electrical circuits, their application in everyday objects is limited.

Several research groups have attempted to identify objects or surfaces using various methods without per-object instrumentation [23,24,25,26,27,28]. For example, ViBand [26] uses a smartwatch to understand the bio-acoustic signals generated by the objects in contact and transmitted through the hand. While ViBand is highly accurate, its application is restricted to objects that generate vibrations, such as an acoustic guitar, a coffee grinder, and a table fan. A device equipped with a vibration motor and inertial sensors, such as a smartphone, can recognize the type of objects in contact by generating vibrations with a certain frequency and analyzing the corresponding readings of the accelerometer [24,25]. Although such devices demonstrate considerable test accuracy, of about 85%, the responses to every frequency elements, such as impulse response, should be observed for better performance. This is because ideally, a system, especially a linear time invariant system, can be completely characterized by its impulse response. Several approaches have also been investigated based on impact made or knock by the user. BeatIt [27] uses a microphone-enabled smartwatch to categorize objects based on the sound generated from the user’s knock. Similarly, Knocker [28] can successfully identify various everyday objects using sound, acceleration, and angular velocity signals. However, these methods require inevitable efforts to detect a valid knock and can hardly be applied to other smart objects, such as AI speakers, because such devices are unable to perform knock actions on their own. Contrary to existing methods, our system is self-aware of the surface on which the device is placed and is highly accurate as it is based on impulse responses.

## 3. Proposed System

In this section, we describe the developed hardware prototype to collect a time-series dataset, and experiments that classify the type of surfaces on which the prototype was placed. We built a surface type-sensing pipeline to evaluate the proposed system. The pipeline primarily comprised of data collection, preprocessing, and machine learning.

### 3.1. Hardware

**Figure 1:**
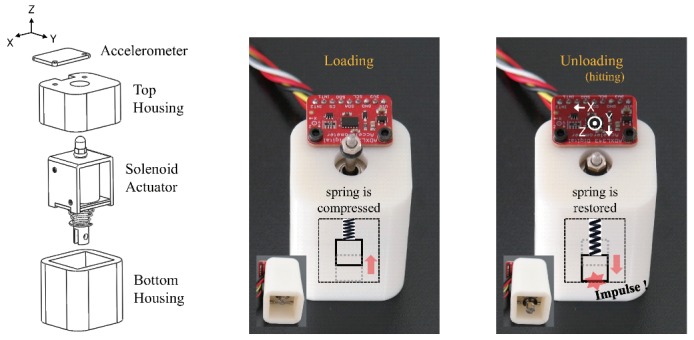
Prototype for knocking the surface and listening to the vibrations. We placed the solenoid actuator inside a 3D-printed housing and mounted an accelerometer on top of the housing. The coordinate system shown in the figure indicates the orientation of the accelerations.

**Figure 2:**
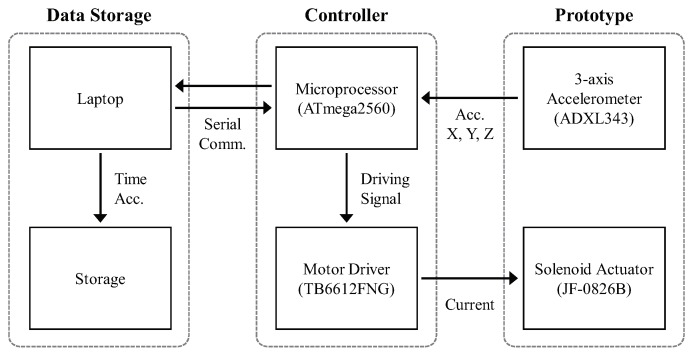
Block diagram of the entire system for data acquisition. We used inter-integrated circuit (I2C) communication to transmit the signals captured by the accelerometer to the microprocessor.

First, we built a prototype comprising a solenoid actuator (JF-0826B, Yueqing gangbei Electric, Wenzhou, China), three-axis accelerometer (ADXL343, Analog devices, Norwood, MA, USA), and three-dimensional (3D) printed housing parts, as shown in Figure 1. The solenoid actuator included a large coil of copper wire with an armature (a metal slug) in the middle. When an electric current was applied to the coil, the slug was pulled to the center of the coil (i.e., loading motion) compressing an elastic spring. When the electric current was removed, the slug was moved down (i.e., hitting the ground) due to the elastic restoring force (see Supplementary Video for more detail). The solenoid actuator used in this study measured 27 mm × 25 mm × 23 mm (the body), and 7.5 mm × 54 mm (the metal slug). We attached an accelerometer onto the housing of the prototype to measure the acceleration signals caused by the loading and hitting motions of the solenoid actuator. The sensor had three axes of measurements (X, Y, and Z), and a range of ±16 g was set for each axis. Figure 2 shows the block diagram of the constructed hardware setup to collect a dataset. The setup comprised the aforementioned prototype, ATmega2560 microprocessor (Arduino Mega), motor driver (TB6612FNG, Toshiba, Tokyo, Japan), and laptop. When the solenoid actuator was driven by a signal amplified by the motor driver from the microprocessor, the captured acceleration signals were transmitted to the laptop via a serial communication and then saved.

### 3.2. Dataset

**Figure 3:**
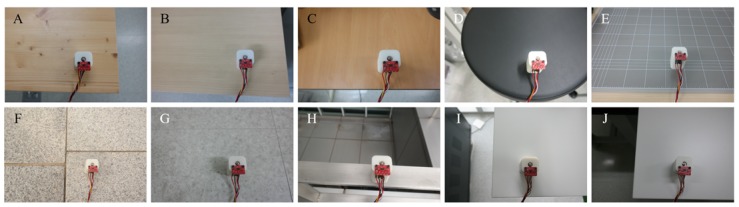
Ten different daily surfaces explored in this study. (**A**): Softwood table, (**B**): Synthetic wood table, (**C**): Plywood table, (**D**): Polyurethane chair, (**E**): Cutting mat, (**F**): Granite tile, (**G**): Porcelain tile, (**H**): Metal plate, (**I**): ABS (acrylonitrile butadiene styrene) plastic table, (**J**): Laminate table.

**Figure 4:**
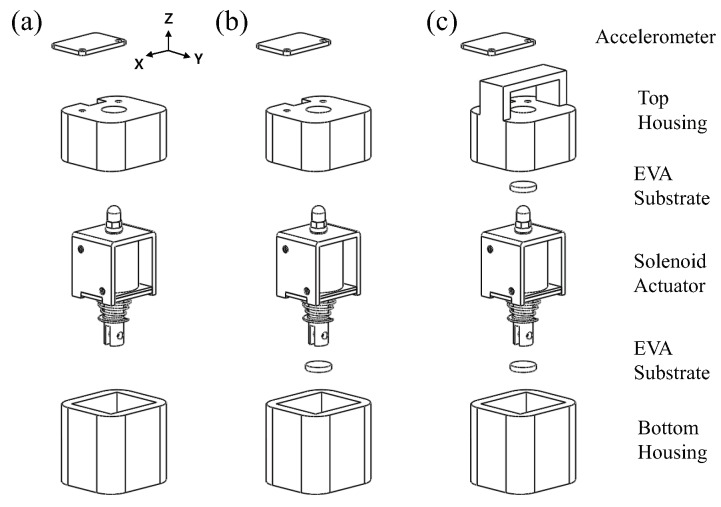
Three different hardware configurations used to collect the data samples. (**a**) The configuration described in Figure 1. We attached an ethylene vinyl acetate (EVA) substrate to (**b**) the bottom of the slug and (**c**) to the top and bottom of the slug, to diversify the characteristics of the loading motion, unloading motion, and resulting responses.

**Figure 5:**
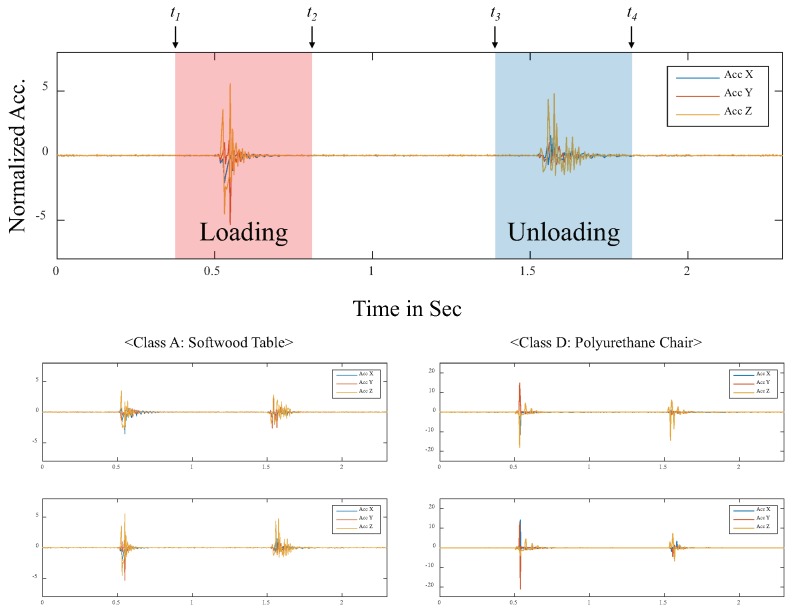
Examples of sample data. Signals highlighted in red (left) were captured while pulling the slug (loading); signals highlighted in blue (right) were captured while the slug crashed onto the desktop surface (unloading or hitting).

We considered a 10-class classification problem. Ten types of daily surfaces (substrates) were prepared to collect time-series data, as shown in Figure 3. To reduce the dependency on a specific hardware, we collected 2000 data samples for three configurations each, translating into a total of 6000 data samples per class, as shown in Figure 4. To avoid overfitting to any specific mechanical structure of each test surface, the data samples were collected from various, randomly selected locations on the test surfaces. Furthermore, objects placed around the prototype were randomly changed during the data collection to reduce the effect of the surroundings. In total, 60,000 data samples (6000 samples per class) were collected. Each sample comprised 600 time steps and were captured at 250 Hz (approximately 2.4 s). Figure 5 shows an example of the collected data sample plotted in the time domain. The horizontal and vertical axes represent time and normalized acceleration, respectively. After 0.5 s from the start of data collection, the slug of the solenoid actuator was pulled up (loading). Subsequently, after 1.0 s, the slug was released to hit the surface (unloading).

During loading/unloading motion, the prototype was swayed in both the vertical (z axis) and horizontal (x and y axes) directions. Although the measured signals in each axis were different for every trial, the overall behavior appeared to be similar for each surface. We focused on the following three windows; (i) loading: 100 time steps near loading motion of the solenoid actuator ($t_{1}$ to $t_{2}$), (ii) unloading: 100 time steps near unloading motion ($t_{3}$ to $t_{4}$), and (iii) loading & unloading: 360 time steps including both loading and unloading motions ($t_{1}$ to $t_{4}$).

The datasets were split into two independent sets: A training set and a test set. Seventy percent of the total dataset were randomly chosen and used for training (4200 samples per class), and the remainder of the dataset was used for testing (1800 samples per class). All the captured acceleration signals were normalized prior to conversion into the input representations for machine learning models. These normalized accelerations were directly used as input representations for one-dimensional convolutional neural network (1D-CNN), long short-term memory (LSTM), and gated recurrent units (GRU) classifiers.

### 3.3. Machine Learning

#### 3.3.1. Baseline Classifier

As a baseline, we adopted a typically used classification method, i.e., the random forest (RF) classifier, which does not consider the sequential characteristics of a given signal [29,30]. For feature engineering, we calculated the following 11 features based on each of three sequential measurements (i.e., normalized three-axis accelerations) and total acceleration: Mean, median, min, max, max/min, std, skew, abs_min, abs_max, abs_mean, abs_std (See Table 1 for detail). Hence, 44 (four measurements × 11 features) features were used for the RF classifier.

#### 3.3.2. One-Dimensional CNN (1D-CNN) Model

**Figure 6:**
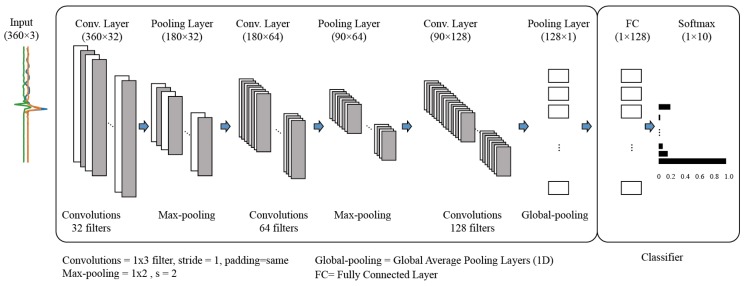
One-dimensional convolutional neural network (1D-CNN) models used in this study (for input data of 360 time steps, loading & unloading window). We used the same architecture for the input data of 100 time steps (loading and unloading windows); however, the input and output shapes were slightly different. Rectified linear function (ReLU) was used as activation function except for the output node that uses a softmax function.

CNNs are widely used in many modern time-series applications owing to their capability in learning both local and global features from sequential data [21,31,32,33]. In this study, to create a 1D-CNN model, we employed a sequential model implemented in Keras. The model structure used in this study is shown in Figure 6. We stacked three consecutive convolutional layers with three pooling layers. A dense layer with a 25% dropout rate was positioned at the end of the network followed by a softmax layer.

#### 3.3.3. Gated RNNs—LSTM and GRU

**Figure 7:**
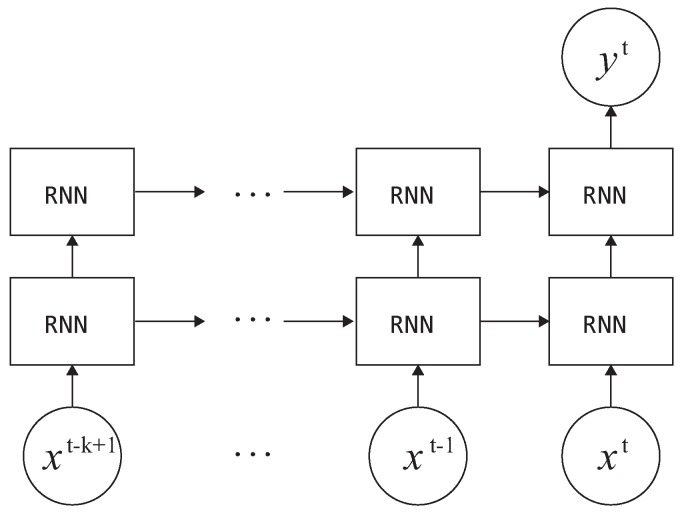
Recurrent neural network (RNN)-based architecture used for the proposed surface type classification. In this study, we employed long short-term memory (LSTM) and gated recurrent units (GRU) cells.

With advancements in recurrent neural networks (RNNs), studies on sequence classification have been actively conducted. Traditional RNNs, which use simple recurrent neurons, have caused problems such as exploding or vanishing gradients [34,35]. Therefore, RNNs were difficult to train through backpropagation. In recent approaches, existing simple neuronal structures have been modified using memory cells and gate units to more efficiently learn dependencies over longer intervals [35,36,37,38]. In this study, we evaluate the performance of the proposed floor type classification using two such neural networks, namely long short-term memory (LSTM) and gated recurrent units (GRUs). The RNN-based architecture used in this study is shown in Figure 7. A dense layer with 50% dropout rate was positioned at the end of the network followed by the a softmax layer.

## 4. Evaluation

**Figure 8:**
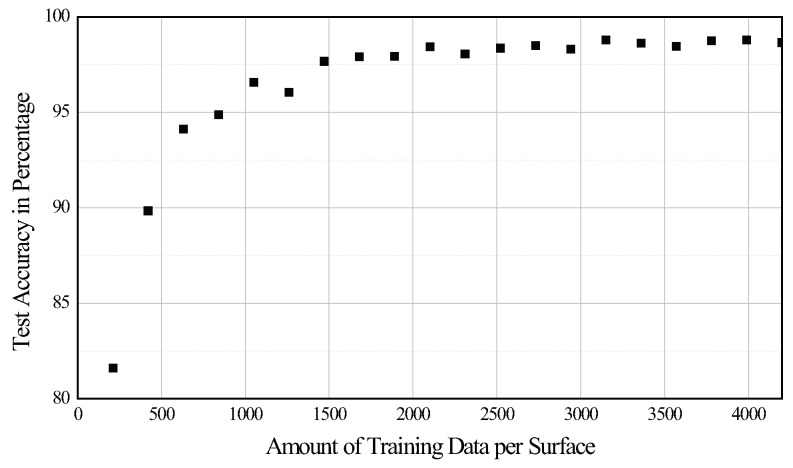
Test accuracy as a function of the amount of training data per surface, using the 1D-CNN classifier.

In this section, we report the classification performance, in terms of test accuracy and f-measure, using four methods described in previous sections (i.e., RF, 1D-CNN, LSTM, and GRU). The test accuracy was calculated as the ratio of the total correct predictions to the total number of input samples (test data of 18,000 instances) without weight, because the dataset was completely balanced. All the classifiers were trained and validated for 21 different cases (seven cases of input signals × three windows) individually. Here, the input signal denotes the axes of the acceleration signals (or channels) used as the input representation to the classifier. Table 2 summarizes the experimental results. The RF classifier performed significantly worse than other classifiers (i.e., 1D-CNN, LSTM, and GRU) because it did not consider the sequential nature of the given dataset. Additionally, we observed that the higher the dimension of the input signals, the better is the performance of the classifier. Notably, 10 different classes were reliably classified even when only one (95.28%, GRU, acc *Z* with loading & unloading window) or two input signals (98.56%, 1D-CNN, acc YZ with loading & unloading window) were used. On the one hand, a better performance was achieved when the input signals included both the loading and unloading motion of the solenoid actuator (loading & unloading windows) than otherwise (loading or unloading windows). On the other hand, it is noteworthy that the results exhibited reasonable performance (97.51%) even when the input signals only included loading motion (e.g., LSTM, acc XYZ with loading window). We further investigated the test accuracy according to the amount of training data per surface, as shown in Figure 8. As expected, the test accuracy increased with the increase in the amount of training data. In particular, 420 data samples per surface achieved an accuracy of approximately 90%. With 840 samples per surface, the proposed approach reached 95% accuracy.

**Figure 9:**
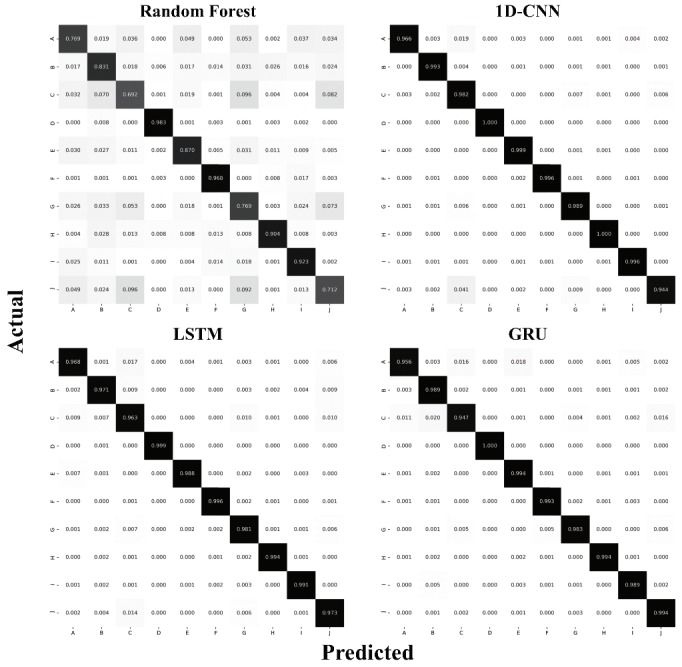
Confusion matrices of the classification results across all 10 class categories for the experiments using three input signals (X, Y, and Z) with loading & unloading window. The majority of misclassifications were between classes C (plywood wood table) and J (laminate table).

**Figure 10:**
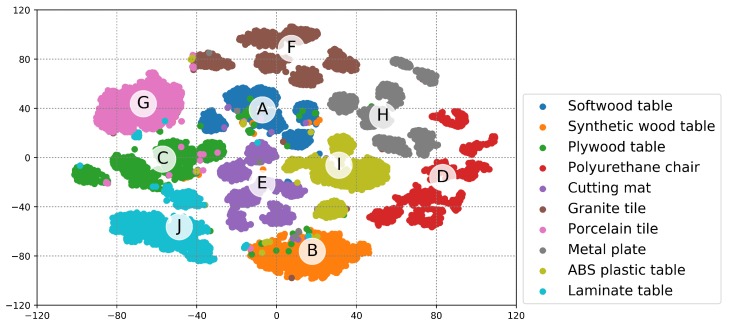
An example of 2-D embedding of the network output of the test dataset used in this study using t-distributed stochastic neighbor embedding (t-SNE) [39] when GRU-based architecture was employed for test.

Figure 9 shows the confusion matrices across all 10 class categories for the experiments using three input signals (X, Y, and Z) with loading & unloading window. More misclassifications occurred between the classes in the similar material group than between different groups (especially, between class C (plywood wood table) and J (laminate table)). Nevertheless, the resulting test accuracy validated by the LSTM was greater than 96% for all classes. Figure 10 shows an embedding of the network output projected into 2D space for visualization using the t-distributed stochastic neighbor embedding (t-SNE) algorithm [39]. Because features learned in a high-dimensional space are separable as Figure 10 shows, we can conclude that the proposed approach is feasible.

## 5. Discussion

### 5.1. General Discussion

Our system could sense the surface type even when only a few inertial measurements (e.g., one or two axes of accelerations) were used as the input signal. This would reduce multiply-accumulates of the system for high-speed/low-power inference operation. Meanwhile, remarkably, the proposed system could classify surface types using only signals captured by the loading motion (i.e., without directly hitting the surface). In fact, it is not desired to directly hit (or tap) a surface, especially one that is fragile or prone to wounds. Therefore, that the properties of a surface can be identified only by internal impact (i.e., loading motion) without directly hitting the surface is an important finding. This will allow the proposed system to be expanded to various application fields. In this study, we focused on identifying the feasibility of the proposed system rather than on maximizing the performance of each classifier. As shown in the previous sections, we devoted only a little effort on the preprocessing of the dataset and employed relatively simple network architectures. In our opinion, the performance of the proposed system can be further enhanced by data augmentation and fine-tuning of the network structure.

Overall, the proposed system successfully identified various surface types, even for surfaces of similar materials. Furthermore, the results of using fewer input signals (one- or two-axes) or using only the loading motion exhibited considerable test accuracy. As such, we have identified the feasibility of predicting the surface type by simply knocking on the surface. We expect these results to provide insight into the investigation of the surrounding environment.

### 5.2. Scalability

The hardware setup is of a simple configuration, and the components used in this study are relatively inexpensive. Therefore, the setup can be easily replicated. In particular, it can be implemented with low-cost computing systems (or embedded systems) because it does not require a high sampling rate and can achieve a reasonable performance with only a few input signals and simple network structure. Furthermore, because surface properties can be determined without directly hitting the surface (i.e., by simply lifting a slug that touched the surface), they are applicable to most surfaces used daily as well as fragile or impact-prone surfaces. In summary, because the proposed system is inexpensive and has a simple configuration, it can be easily replicated or embedded into a wide range of smart commodities such as AI speakers, mobile robots, and vacuum cleaning robots. Meanwhile, for practical applications, data collection process might be onerous. As shown in Figure 8, the test accuracy reached 90% with 420 data samples per surface. In the proposed system, it takes approximately 17 min to collect 420 samples. We believe that this is not a huge burden to extending our approach to new surfaces of interest.

### 5.3. Context Understanding Based on Location

**Figure 11:**
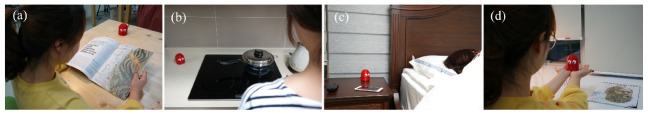
Applications of the proposed knocking and sensing strategy in various interaction scenarios: AI speaker interacting with a user on the desk (**a**), on the kitchen table (**b**), in the bed room (**c**), and on the palm of the hand (**d**).

Owing to the controversy around breach of privacy with regard to camera-based smart devices, they are not widely used in home appliances, despite the advantage that cameras can obtain considerable contextual information around the device. As a result, most AI speakers sold in the market are not accompanied by a camera. Although voice recognition-based AI speakers can interact with users in a variety of scenarios, the lack of contextual information limits their applications. An example of contextual information that could be provided by a smart device is its location, which could be the bedroom, kitchen, living room, etc. To elaborate further, if an AI speaker can sense that it is placed on the table of a kitchen, it could talk to the user in the context of food, such as ordering cooking supplies to prepare dinner. Additionally, if the AI speaker is placed near a user reading a book at a desk, it might recommend proper lighting conditions. Figure 11 shows examples of such interaction scenarios.

### 5.4. Limitations and Future Work

In this study, we attempted to prevent trained models from being overfitted to specific hardware by using three prototypes of different configurations to collect data. In addition, to reduce the structural effect of the surface, we collected data from various locations on each test surface. As a result, we determined the feasibility of classifying different types of surfaces by using the proposed system. However, if the proposed system is embedded into a large device, such as an AI speaker, it may not be able to use the currently trained model as it is. The system may require additional data collected from the target device configuration. Note that this study focused on verifying the feasibility of the proposed system at the prototype level. In a future study, we will collect additional data with more diverse hardware configurations and extend the proposed system to a variety of smart devices.

To reduce the structural effect (or geometric property) of the surfaces, we collected the dataset from various locations on each test surface with random objects placed around the prototype. However, some geometric properties might be learned by the machine learning model. Thus, at the current stage, it is difficult to say that the proposed method is a universal approach for characterizing surface materials. However, we observed the possibility that the proposed method can identify generic material properties. For example, our system could discern classes F and G (granite-tiled floor and porcelain-tiled floor). As they have almost the same geometry and boundary conditions, we believe that our approach can be extended to classify generic surface characteristics. To construct a robust and generalized model, we plan to conduct additional experiments in various surfaces made of the same material but different shapes and boundary conditions.

In our current design, the most significant drawback is the inevitable physical impact. As mentioned in the discussion section, the proposed system demonstrated reasonable performance even when only the loading motion was used as the input to the classifier. However, to produce a loading motion, an unloading motion must be proceeded, and it was difficult to control the system to hit the surface weakly owing to the intrinsic operating principle of the solenoid actuator. Furthermore, the solenoid actuator used in this study measured 27 × 25 × 23 mm (the body), and 7.5 × 54 mm (the metal slug), which might not be suitable for embedment into small electronic devices (or robots). These aspects would hinder the potential applications of the proposed system. In the future, we plan to (1) further improve the performance using only the loading motion, (2) adopt weak loading and unloading motions that do not damage the surface or produce no audible noise, (3) modify the mechanism to be compact instead of using a bulky actuator.

## 6. Conclusions

In this paper, we proposed an intelligent system to predict surface type by analyzing multivariate time-series sensor signals captured by mechanically knocking a surface. First, the hardware setup (prototype) was constructed, and the dataset (60,000 samples in total) was collected from 10 different test surfaces using the prototype. Subsequently, we designed a series of classifiers such as RF, CNN, and gated RNNs (LSTM and GRU). The performance was evaluated in terms of test accuracy and f-measure. Overall, the proposed system successfully classified 10 different surface types (accuracy of 98.73% with GRU). Notably, a test accuracy of 96.53% was achieved even when using only signals captured when the slug of the solenoid actuator was lifted off the surface (i.e., without hitting the surface directly). We expect our approach to be expandable to various applications including AI speakers and mobile robots, as the proposed system is compact and does not require expensive components; thus, it can be easily replicated or embedded into other devices for contextual interaction.

## Figures and Tables

**Table 1:** Features explored for random forest classifier. Eleven features were calculated based on each of the three-axis acceleration measurements.

Feature	Descriptions
mean	arithmetic mean (average)
median	median
min	minimum
max	maximum
max/min	ratio of max and min
std	standard deviation
skew	sample skewness
abs_min	minimum of absolute value
abs_max	maximum of absolute value
abs_mean	arithmetic mean of absolute value
abs_std	standard deviation of absolute value

**Table 2:** Experimental results—test accuracy in percentage. The values in parentheses denote the f-measure score. Ten classes were reliably classified even when only one or two input signals were used. Notably, the results exhibited reasonable performance even when using only the loading motion (loading window).

Input Signals	Window	RF	1D-CNN	LSTM	GRU
Acc. X	loading	42.90 (0.430)	93.10 (0.832)	81.28 (0.813)	79.77 (0.798)
Acc. Y	loading	36.88 (0.368)	81.39 (0.814)	79.74 (0.798)	76.63 (0.769)
Acc. Z	loading	43.19 (0.427)	86.00 (0.859)	87.75 (0.877)	85.41 (0.854)
Acc. X, Y	loading	66.63 (0.666)	92.09 (0.921)	89.96 (0.900)	86.86 (0.869)
Acc. X, Z	loading	67.15 (0.671)	94.52 (0.945)	94.89 (0.949)	93.22 (0.932)
Acc. Y, Z	loading	60.32 (0.602)	93.68 (0.937)	95.57 (0.956)	93.97 (0.940)
Acc. X, Y, Z	loading	75.79 (0.758)	96.38 (0.964)	97.51 (0.975)	96.37 (0.964)
Acc. X	unloading	40.07 (0.399)	77.58 (0.777)	78.04 (0.781)	77.90 (0.778)
Acc. Y	unloading	34.71 (0.348)	75.13 (0.751)	76.38 (0.764)	73.59 (0.738)
Acc. Z	unloading	51.82 (0.516)	87.29 (0.873)	88.18 (0.882)	87.83 (0.879)
Acc. X, Y	unloading	63.99 (0.638)	90.33 (0.904)	90.59 (0.906)	88.74 (0.887)
Acc. X, Z	unloading	72.61 (0.725)	95.26 (0.953)	95.22 (0.952)	94.64 (0.946)
Acc. Y, Z	unloading	69.06 (0.689)	94.62 (0.946)	94.57 (0.946)	93.28 (0.933)
Acc. X, Y, Z	unloading	78.34 (0.782)	96.91 (0.969)	97.57 (0.976)	96.82 (0.968)
Acc. X	both	46.33 (0.462)	87.82 (0.878)	78.36 (0.785)	86.81 (0.868)
Acc. Y	both	37.77 (0.381)	88.25 (0.882)	78.21 (0.781)	83.17 (0.833)
Acc. Z	both	48.78 (0.482)	95.19 (0.952)	93.78 (0.938)	95.28 (0.953)
Acc. X, Y	both	73.04 (0.730)	96.11 (0.961)	93.67 (0.937)	94.04 (0.943)
Acc. X, Z	both	79.09 (0.729)	98.39 (0.984)	97.19 (0.972)	98.21 (0.982)
Acc. Y, Z	both	68.92 (0.689)	98.56 (0.986)	97.28 (0.973)	98.07 (0.981)
Acc. X, Y, Z	both	84.21 (0.842)	98.66 (0.987)	98.23 (0.982)	98.39 (0.984)

## Supplementary Materials

The following are available at https://www.mdpi.com/1424-8220/20/2/369/s1.
